# What is *Nutrition & Metabolism*?

**DOI:** 10.1186/1743-7075-1-1

**Published:** 2004-08-17

**Authors:** Richard D Feinman, M Mahmood Hussain

**Affiliations:** 1 Department of Biochemistry, State University of New York Downstate Medical Center, Brooklyn, NY 11203 USA; 2 Department of Anatomy and Cell Biology, State University of New York Downstate Medical Center, Brooklyn, NY 11203 USA

## Abstract

A new Open Access journal, *Nutrition & Metabolism *(*N&M*) will publish articles that integrate nutrition with biochemistry and molecular biology. The open access process is chosen to provide rapid and accessible dissemination of new results and perspectives in a field that is of great current interest. Manuscripts in all areas of nutritional biochemistry will be considered but three areas of particular interest are lipoprotein metabolism, amino acids as metabolic signals, and the effect of macronutrient composition of diet on health. The need for the journal is identified in the epidemic of obesity, diabetes, dyslipidemias and related diseases, and a sudden increase in popular diets, as well as renewed interest in intermediary metabolism.

## Editorial

Recent events that provide the rationale for a new Open Access journal, *Nutrition & Metabolism *(*N&M*) include 1) an awareness of an epidemic of obesity, diabetes, dyslipidemias and related diseases, 2) a sudden increase in the popularity of diets, such as low carbohydrate diets, to achieve weight loss and combat diabetes, and 3) a renewed interest in intermediary metabolism accompanied by the development of new tools and techniques for genomic and metabolic analysis.

With the considerable activity shown in these areas, rapid and easily accessible dissemination of new information is clearly valuable. Whereas articles in existing journals do discuss intermediary metabolism in a nutritional context, there is a need for a unique and explicit focus for this discipline. In addition, it is precisely because publications in nutritional biochemistry are spread over such a large number of existing journals, few libraries and almost no individual can subscribe to all. It is in areas like this that free, open access becomes important. There is a large published debate on open access (see, e.g. [1]). Most recently, the UK House of commons issued a report encouraging open access publishing of government-funded research (available with comments through ) and similar motions exist in the US congress [2,3]. The editors of *N&M *feel that, at this point, the burden of proof is on proponents of perpetuating the current system. We are, however, not doctrinaire on this point and believe one should pay for a service if it is valuable. Beyond information, printed collections provide convenience and we intend to offer bound copies of articles on individual topics as the journal proceeds.

Nutrition and metabolism is a broad field and we welcome submissions from all areas of nutrition and related biochemistry. Like any journal, however, *N&M *has its own strengths and interests as indicated by the board of editors . Three areas of particular interest are lipoprotein metabolism, amino acids as metabolic signals, and the effect of macronutrient composition of diet on health. This is reflected in our opening research articles by Darimont, *et al*. on the control of obesity and lipid structure by adrenergic systems, and by Volek, *et al*. on the effectiveness of low carbohydrate diets, and differential effects on fat and lean mass.

The sudden popularity of low carbohydrate diets is one of the most remarkable phenomena in nutrition today. A recent editorial by Walter Willett points out how important it is that we understand them [4]. Similarly, the recent conference on Nutritional and Metabolic Aspects of Low Carbohydrate Diets , while not recommending any particular diet, highlighted many of the relevant issues in macronutrient control of metabolism. In our initial publications, contributors to the conference will provide reviews of the various topics covered. In the first posting, Klaas Westerterp summarizes the importance of macronutrient composition in thermogenesis, and Stephen Phinney discusses the impact of ketogenic diets on physical performance. Kimball and Jefferson review the regulation of mRNA translation in general. The article provides a nice overview of various mechanisms involved in the control of protein synthesis when amino acids become limiting. Perhaps the most important from a practical standpoint, Nuttall and Gannon summarize potential benefits of higher protein diets in diabetes.

Nutrition & Metabolism welcomes contributions in all areas of research in which nutrition interacts with biochemistry and molecular biology. Emphasis will be on the molecular, biochemical, and physiologic understanding of various metabolic pathways. The journal will publish Original Research, Reviews, Commentaries and Perspectives, Brief Communications, Methods and Book Reviews. Access to all articles in *N&M *is free. Articles are included in PubMed and archived in PubMed Central. Online submissions can be made at .
